# Market landscape of penicillin for syphilis and rheumatic heart disease, WHO South-East Asia Region

**DOI:** 10.2471/BLT.25.295053

**Published:** 2026-06-03

**Authors:** Gereltuya Dorj, Uhjin Kim, Po-Lin Chan, Pradeep Joshi, Abhishek Royal, Karina Razali

**Affiliations:** a National Drug and Alcohol Research Centre, Faculty of Medicine & Health, University of New South Wales Sydney, 55 Botany St, Sydney, New South Wales, 2031, Australia.; b Access to Medicines, Technologies, Diagnostics and Health Products, Regional Office for South-East Asia, World Health Organization, New Delhi, India.; c Hepatitis, HIV, Sexually Transmitted Diseases and Tuberculosis, Regional Office for South-East Asia, World Health Organization, New Delhi, India.; d Noncommunicable Diseases, Mental Health and Disabilities, Regional Office for South-East Asia, World Health Organization, New Delhi, India.; e World Health Organization, Sub-office Cox's Bazar, Bangladesh.

## Abstract

**Objective:**

To assess the market landscape of penicillin formulations used for the treatment of syphilis and rheumatic heart disease in the World Health Organization South-East Asia Region.

**Methods:**

We analysed market data for five essential penicillin formulations across nine countries in the South-East Asia Region using a survey form to collect data on product registration, inclusion in national essential medicines lists, procurement, availability, pricing and demand forecasting.

**Findings:**

Substantial disparities exist in registration status, procurement and pricing across the region. The price of benzathine benzylpenicillin 1.2 million international units (IU) in Timor-Leste was over 23 times higher than in Bangladesh (3.45 United States dollars, US$, versus US$ 0.15), while the price for 2.4 million IU varied by up to 55–fold across the region. Several countries reported supply issues, including shortages of benzathine benzylpenicillin in India, Maldives, Myanmar, Thailand and Timor-Leste; aqueous benzylpenicillin in Sri Lanka during the 2022 economic crisis; and supply disruptions of phenoxymethylpenicillin 250 mg in Maldives and Sri Lanka. Morbidity-based estimates indicated annual regional demand of 171 966 061 vials of benzathine benzylpenicillin 2.4 million IU and 923 053 121 tablets of phenoxymethylpenicillin 250 mg, with corresponding costs of US$ 62 127 441 and US$ 48 005 181, respectively. India has the highest demand of these two formulations.

**Conclusion:**

Differences in registration, procurement, pricing and forecasting undermine reliable access to penicillin products in the South-East Asia Region. Stronger regional collaboration, improved forecasting, harmonized regulatory approaches and pooled procurement strategies are needed to secure equitable and sustainable access.

## Résumé

**Objectif:**

Évaluer le panorama du marché des préparations à base de pénicilline utilisées pour le traitement de la syphilis et des cardiopathies rhumatismales dans la région de l’Asie du Sud-Est de l’Organisation mondiale de la Santé.

**Méthodes:**

Nous avons analysé les données de marché relatives à cinq préparations essentielles à base de pénicilline dans neuf pays de la région de l’Asie du Sud-Est, à l’aide d’un questionnaire permettant de recueillir des informations sur l’homologation des produits, leur inclusion dans les listes nationales de médicaments essentiels, leur approvisionnement, leur disponibilité, leur tarification et les prévisions de demande.

**Résultats:**

Il existe des disparités importantes en matière d’homologation, d’approvisionnement et de tarification à l’échelle de la région. Le prix de la benzylpénicilline benzathine à 1,2 million d’unités internationales (UI) au Timor-Leste était plus de 23 fois supérieur à celui pratiqué au Bangladesh (3,45 dollars américains (USD), contre 0,15 USD), tandis que le prix pour 2,4 millions d’UI variait jusqu’à 55 fois dans la région. Plusieurs pays ont signalé des problèmes d’approvisionnement, notamment des pénuries de benzylpénicilline benzathine en Inde, aux Maldives, au Myanmar, en Thaïlande et au Timor-Leste; de benzylpénicilline aqueuse au Sri Lanka pendant la crise économique de 2022; et des perturbations de l’approvisionnement en phénoxyméthylpénicilline à 250 mg aux Maldives et au Sri Lanka. Les estimations fondées sur la morbidité ont mis en évidence une demande régionale annuelle de 171 966 061 flacons de benzylpénicilline benzathine à 2,4 millions d’UI et de 923 053 121 comprimés de phénoxyméthylpénicilline à 250 mg, pour des coûts correspondants de 62 127 441 USD et 48 005 181 USD. C’est en Inde que la demande pour ces deux préparations est la plus forte.

**Conclusion:**

Les différences en matière d’homologation, d’approvisionnement, de tarification et de prévisions compromettent la fiabilité de l’accès aux préparations à base de pénicilline dans la région de l’Asie du Sud-Est. Le renforcement de la collaboration régionale, l’amélioration des prévisions, l’harmonisation des approches réglementaires et le regroupement des stratégies d’approvisionnement sont nécessaires pour garantir un accès équitable et durable.

## Resumen

**Objetivo:**

Evaluar el panorama del mercado de las formulaciones de penicilina utilizadas para el tratamiento de la sífilis y la cardiopatía reumática en la Región de Asia Sudoriental de la Organización Mundial de la Salud (OMS).

**Métodos:**

Se analizaron datos de mercado correspondientes a cinco formulaciones esenciales de penicilina en nueve países de la Región de Asia Sudoriental mediante un cuestionario para recopilar información sobre el registro de los productos, su inclusión en las listas nacionales de medicamentos esenciales, la adquisición, la disponibilidad, los precios y la previsión de la demanda.

**Resultados:**

Existen importantes diferencias en el estado del registro, la adquisición y los precios entre los países de la región. El precio de la bencilpenicilina benzatina de 1,2 millones de unidades internacionales (UI) en Timor-Leste fue más de 23 veces superior al de Bangladesh (3,45 dólares estadounidenses [US$] frente a US$ 0,15), mientras que el precio de la presentación de 2,4 millones de UI varió hasta 55 veces entre los países de la región. Varios países notificaron problemas de suministro, entre ellos la escasez de bencilpenicilina benzatina en India, Maldivas, Myanmar, Tailandia y Timor-Leste; de bencilpenicilina sódica acuosa en Sri Lanka durante la crisis económica de 2022; y las interrupciones en el suministro de fenoximetilpenicilina de 250 mg en Maldivas y Sri Lanka. Las estimaciones basadas en la morbilidad indicaron una demanda anual regional de 171 966 061 viales de bencilpenicilina benzatina de 2,4 millones de UI y 923 053 121 comprimidos de fenoximetilpenicilina de 250 mg, con unos costes correspondientes de US$ 62 127 441 y US$ 48 005 181. India presenta la mayor demanda de estas dos formulaciones.

**Conclusión:**

Las diferencias en el registro, la adquisición, los precios y la previsión de la demanda comprometen un acceso fiable a los productos de penicilina en la Región de Asia Sudoriental. Se requiere una mayor colaboración regional, una mejor previsión de la demanda, enfoques regulatorios armonizados y estrategias de adquisición conjunta para garantizar un acceso equitativo y sostenible.

## ملخص

**الغرض:**

تقييم أوضاع السوق لمستحضرات البنسلين المستخدمة في علاج الزهري وأمراض القلب الروماتيزمية في منطقة جنوب شرق آسيا التابع لمنظمة الصحة العالمية.

**الطريقة:**

قمنا بتحليل بيانات السوق لخمسة مستحضرات أساسية من البنسلين في تسع دول في منطقة جنوب شرق آسيا، باستخدام نموذج استبيان لجمع بيانات حول تسجيل المنتجات، وإدراجها في قوائم الأدوية الأساسية الوطنية، وتوريدها، وتوافرها، وتسعيرها، والتنبؤ بالطلب عليها.

**الاستنتاج:**

توجد فروقات ملموسة في حالة التسجيل، والتوريد، والتسعير في جميع أنحاء المنطقة. كان سعر بنزاثين بنزيل بنسلين بتركيز 1.2 مليون وحدة دولية (IU) في وتيمور - لشتي أعلى بأكثر من 23 ضعفًا من سعره في بنغلاديش (3.45 دولار أمريكي مقابل 0.15 دولار أمريكي)، بينما تفاوت سعر تركيز 2.4 مليون وحدة دولية بما يصل إلى 55 ضعفًا في جميع أنحاء المنطقة. أبلغت عدة دول عن مشاكل في الإمدادات، بما في ذلك نقص بنزاثين بنزيل بنسلين في الهند وملديف وميانمار وتايلند وتيمور - لشتي؛ ونقص بنزيل بنسلين المائي في سري لانكا خلال الأزمة الاقتصادية عام 2022؛ وحالات انقطاع إمداد فينوكسي ميثيل بنسلين 250 ملج في ملديف وسري لانكا. أشارت التقديرات المستندة على معدلات الإصابة إلى طلب إقليمي سنوي يبلغ 171966061 قارورة من بنزاثين بنزيل بنسلين، 2.4 مليون وحدة دولية و923053121 قرصًا من فينوكسي ميثيل بنسلين 250 ملج، بتكاليف مقابلة تبلغ 62127441 دولارًا أمريكيًا، و48005181 دولارًا أمريكيًا على التوالي. وتُعد الهند الدولة الأكثر طلبًا على هذين النوعين من الأدوية.

**الاستنتاج:**

إن الاختلافات في التسجيل والتوريد والتسعير والتنبؤ، تقلل من إمكانية الحصول على منتجات البنسلين بشكل موثوق في منطقة جنوب شرق آسيا. هناك حاجة إلى تعزيز التعاون الإقليمي، وتحسين التنبؤ، وتوحيد الأساليب التنظيمية، واعتماد استراتيجيات توريد مشتركة لضمان وصول عادل ومستدام إلى هذه المنتجات.

## 摘要

**目的:**

评估世卫组织东南亚地区用于治疗梅毒与风湿性心脏病的青霉素制剂的市场格局。

**方法:**

我们使用一份调查表格收集了有关产品注册、国家基本药物目录纳入情况、采购、药品可及性、定价与需求预测的数据，分析了东南亚地区九个国家五种基本的青霉素制剂的市场数据。

**结果:**

该地区各国在注册状态、采购和定价方面差异悬殊。在东帝汶，120 万国际单位 (IU) 的苄星青霉素的价格比孟加拉国（3.45 美元比 0.15 美元）高出超过 23 倍，而 240 万国际单位的该药的价格在整个该地区各国间相差最高达 55 倍。多个国家报告称存在供应问题，包括印度、马尔代夫、缅甸、泰国、东帝汶都存在苄星青霉素短缺问题；2022 年经济危机期间斯里兰卡出现水性苄青霉素短缺情况；马尔代夫和斯里兰卡 250 毫克青霉素 V 钾供货中断。基于发病率的估测显示，每年该地区的需求量为 240 万国际单位的苄星青霉素 171966061 瓶和 250 毫克的青霉素 V 钾 923053121 片，对应的成本分别是 62127441 美元和 48005181 美元。印度对这两种制剂的需求最高。

**结论:**

药品注册、采购、定价与预测的差异阻碍了东南亚地区稳定获取青霉素产品。如要确保公平且可持续的获取相关产品，必须增强地区协调、完善相关预测、协调监管机制和实施联合集中采购策略。

## Резюме

**Цель:**

Оценить ситуацию на рынке лекарственных форм пенициллина, используемых для лечения сифилиса и ревматической болезни сердца в регионе Юго-Восточной Азии Всемирной организации здравоохранения.

**Методы:**

Авторы проанализировали рыночные данные по пяти основным лекарственным формам пенициллина в девяти странах региона Юго-Восточной Азии, для чего использовалась форма опроса, с помощью которой собирались данные о регистрации препаратов, их включении в национальные перечни основных лекарственных средств, закупках, доступности, ценах и прогнозировании спроса.

**Результаты:**

В пределах региона наблюдаются значительные различия в статусе регистрации, закупках и ценообразовании. Цена на бензилпенициллин бензатина в дозировке 1,2 млн международных единиц (МЕ) в Восточном Тиморе была более чем в 23 раза выше, чем в Бангладеш (3,45 долл. США по сравнению с 0,15 долл. США), в то время как цена препарата в дозировке 2,4 млн МЕ отличалась почти в 55 раз в разных странах региона. В некоторых странах сообщалось о проблемах со снабжением, включая дефицит бензилпенициллина бензатина в Восточном Тиморе, Индии, на Мальдивах, в Мьянме и Таиланде, дефицит водного бензилпенициллина в Шри-Ланке во время экономического кризиса 2022 года, а также перебои в поставках феноксиметилпенициллина в дозировке 250 мг на Мальдивах и в Шри-Ланке. Оценки, основанные на показателях заболеваемости, указали на то, что ежегодная потребность региона составляет 171 966 061 флакон бензилпенициллина бензатина (в дозировке 2,4 млн МЕ) и 923 053 121 таблетку феноксиметилпенициллина (в дозировке 250 мг) стоимостью 62 127 441 долл. США и 48 005 181 долл. США соответственно. Самый высокий спрос на эти две лекарственные формы наблюдается в Индии.

**Вывод:**

Различия в регистрации, закупках, ценообразовании и прогнозировании нарушают надежный доступ к препаратам пенициллина в регионе Юго-Восточной Азии. Необходимы более тесное региональное сотрудничество, улучшенное прогнозирование спроса, гармонизированные регуляторные подходы и внедрение стратегий совместных закупок, чтобы обеспечить справедливый и устойчивый доступ к лекарственным препаратам.

## Introduction

Reliable access to penicillin formulations is crucial for treating congenital syphilis and managing rheumatic heart disease.^1^^,^^2^ Interventions have demonstrated that elimination of mother-to-child transmission of syphilis is attainable when timely diagnosis and treatment are ensured.^3^ Without treatment, syphilis can lead to stillbirth, neonatal death and congenital infection.^4^ The World Health Organization (WHO) recommends injectable benzathine benzylpenicillin (hereafter benzathine penicillin G) as the first-line therapy for maternal syphilis and injectable aqueous benzylpenicillin (hereafter penicillin G) for congenital syphilis;^1^ there are no recommended oral formulations, making reliable access to injectable penicillin essential. For rheumatic heart disease, long-term intramuscular benzathine penicillin G prophylaxis is essential to prevent recurrent rheumatic fever and disease progression, with oral phenoxymethylpenicillin (hereafter penicillin V) recommended only when benzathine penicillin G is not feasible.^2^ All five key penicillin formulations indicated for the treatment and management of these conditions (benzathine penicillin G 1.2 million international units (IU) and 2.4 million IU, penicillin G 1 million IU and penicillin V 250 mg and 500 mg) are included in the *WHO Model list of essential medicines, 24^th^ list.*^5^

Despite these longstanding guidelines recommending penicillin-based regimens, there have been intermittent global penicillin shortages.^6^ In the WHO South-East Asia Region, access is uneven due to fragmented supply chains, regulatory barriers and weak market incentives.^7^ Many countries in the region report inconsistent availability, formulation-specific shortages and fragmented procurement systems.^6^^–^^10^

In the South-East Asia Region, syphilis is a significant public health concern. In 2021, an estimated 22 500 pregnant women reported with syphilis infection, leading to 8100 infants with congenital syphilis and 5000 adverse pregnancy outcomes.^11^ WHO’s triple elimination framework aims to eliminate mother-to-child transmission of human immunodeficiency virus (HIV), syphilis and hepatitis B, but progress across the region is uneven.^3^ Maldives and Sri Lanka have maintained validated elimination status since 2019, whereas Thailand, which achieved validated elimination in 2016, has reported a rising trend in cases in recent years.^12^^–^^15^ Congenital syphilis data across the region remain limited and national reporting practices vary widely (Table 1).

**Table 1:** Country-level burden of syphilis and rheumatic heart disease, WHO South-East Asia Region, 2024–2025

Member State	Population^a^	Syphilis^b^					Rheumatic heart disease	
		Pregnant women tested	Positive pregnant women	Treated pregnant women	Congenital cases		Prevalence per 100 000^c^	Estimated cases
Bangladesh	172 000 000	583 200	0	0	NA		756	1 295 671
Bhutan	780 000	12 000	54	54	49^c^		733	5 765
India	1 428 600 000	20 678 000	8 271	7 055	144		691	9 935 030
Democratic People’s Republic of Korea^d^	26 100 000	1 110	0	0	NA		711	187 756
Maldives	530 000	9 000	0	0	NA		765	4 023
Myanmar	55 200 000	627 300	4 077	3 939	NA		862	466 414
Nepal	30 900 000	136 500	915	296	NA		693	205 638
Sri Lanka	21 600 000	299 840	150	136	14		63	14 458
Thailand	71 800 000	596 400	10 258	9 837	1 290		724	518 908
Timor-Leste	1 400 000	37 480	311	280	NA		787	10 900

NA: not available; WHO: World Health Organization.

^a^ Estimated population for 2024–2025 from United Nations World Population Prospects 2024.^16^

^b^ Syphilis age-standardized data in 2024.^14^

^c^ Rheumatic heart disease prevalence data.^15^

^d^ Democratic People’s Republic of Korea was only included in the forecasting analysis for the region, using published data.^14^^,^^15^ Survey data were not submitted for the broader analyses.

Rheumatic heart disease is also a significant public health issue in the region, driven by socioeconomic inequities including poverty, overcrowding and constrained treatment access.^2^ In 2021, the highest rheumatic heart disease prevalence in the region was reported in Myanmar (862 cases per 100 000 population), Timor-Leste (787 cases per 100 000 population) and Maldives (765 cases per 100 000 population); India, the country with the highest disease burden, reported prevalence of 691 cases per 100 000 population.^15^

Successful treatment and management of syphilis and rheumatic heart disease depend on the reliable availability of penicillin products, which come from the same penicillin supply chain. However, region-specific evidence on how penicillin registration, its inclusion on national essential medicines lists, procurement, pricing and forecasting interact to shape access remains limited.^7^^,^^17^ Here, we comprehensively assess the penicillin market functionality, formulation-specific vulnerabilities and integrated procurement and forecasting needs across the Member States of the WHO South-East Asia Region.

## Methods

### Study design 

This cross-sectional observational study collected data using a structured survey (available from online repository),^18^ which we distributed to national regulatory, procurement and health programme authorities across 10 Member States in the region between 1 February 2025 and 30 May 2025. Nine countries responded: Bangladesh, Bhutan, India, Maldives, Myanmar, Nepal, Sri Lanka, Thailand and Timor-Leste. For data on shortages, availability and quantification practices we used a reporting period of the previous three years (2022 to 2025) and for cost data for penicillin we used costs from the most recent and available reporting period.

### Data collection

The survey focused on the five essential penicillin formulations recommended by WHO for the treatment of syphilis and management of rheumatic heart disease:^5^ benzathine penicillin G 1.2 million IU and 2.4 million IU; penicillin G 1 million IU; penicillin V 250 mg and 500 mg. For each formulation, we collected market and policy data across seven dimensions: (i) product registration status; (ii) inclusion in national essential medicines lists; (iii) procurement mechanisms and requirements; (iv) product availability and shortage history; (v) indications and inclusion in national guidelines; (vi) unit prices; and (vii) demand quantification and surveillance practices. 

We collected available morbidity and cost data to calculate demand forecasts for penicillin formulations across the region. We obtained the morbidity data from official sources, including national statistical offices, WHO’s Global Health Observatory and the Institute for Health Metrics and Evaluation’s global burden of disease results; ^14^^,^^15^ we obtained cost data from health ministries.

#### Definitions

To ensure comparability across countries, we applied the following operational definitions: (i) availability is the confirmed presence of a product within the national public-sector supply chain during the preceding three years; (ii) shortages refer to the inability to meet routine programmatic demand due to constraints in procurement, manufacturing or distribution, as reflected in country-reported routine supply and stock-out events; (iii) sufficiency is the absence of reported stock-outs within a three-year period. 

We classified procurement mechanisms by purchasing authority level: centralized (medicines purchased through a national-level authority); decentralized (purchasing at sub-national or facility level); and mixed (combining centralized procurement with supplementary mechanisms such as sub-national purchasing or external supply channels).

### Analytic approach

#### Market-level analyses

We used a mixed-method descriptive approach, synthesizing the survey data on registration, national essential medicines list inclusion, procurement, availability and shortage history, indications and national guidelines through structured tabular extraction and narrative summaries. We tabulated unit price data (most recent procurement cycle, 2024–2025), summarized demand-related data across countries and formulations and conducted price comparisons in R (R Foundation, Vienna, Austria).

#### Formulation-level analyses

We analysed each formulation separately for demand forecasting, pricing, regulatory assessment and clinical use in line with WHO dosing recommendations and country-specific procurement and reporting practices.^1^^,^^2^ We did not assume operational substitution between vial strengths (e.g. using multiple 1.2 million IU vials in place of a 2.4 million IU vial), as differences in registered strength, procurement systems, dosing practices and pragmatic use may limit interchangeability in practice. 

#### Demand forecasting analysis

We used a combined approach for demand forecasting to enable a comprehensive estimate of penicillin needs for maternal syphilis, congenital syphilis and rheumatic heart disease across the region (Table 2), combining disease burden estimates with WHO-recommended treatment regimens.^1^^,^^2^

**Table 2:** Estimated annual demand of penicillin for syphilis and rheumatic heart disease, WHO South-East Asia Region

Condition	Penicillin formulation (strength)	Dosage	Estimated annual need per case	Calculation assumptions
Syphilis in pregnant women	Benzathine penicillin G (2.4 million IU)	1–3 doses depending on stage	2 doses	Programmes typically plan for 2 doses^a^
Congenital syphilis	Penicillin G (1 million IU)	50 000 IU/kg per intramuscular or intravenous dose every 12h for 7 days, then every 8h for 3 days	23 doses	14 doses (days 1–7) plus 9 doses (days 8–10), 23 doses total for 10 days
Rheumatic heart disease in adults	Benzathine penicillin G (2.4 million IU)	1 intramuscular dose every 3 weeks	17 doses per year	Based on 52 weeks in 3-week interval
Rheumatic heart disease in adults	Penicillin V (250 mg)	250 mg oral dose twice daily	730 tablets per year	2 tablets per day for 365 days
Rheumatic heart disease in children	Penicillin V (250 mg)	250 mg oral dose once daily	365 tablets per year	1 tablet per day for 365 days

IU: international units; WHO: World Health Organization.

^a^ Syphilis calculations assume a mix of early and late syphilis.

Notes: Regimens, dosage and calculation assumptions are based on WHO-recommended treatment regimens.^1^^,^^2^ Benzathine penicillin G and penicillin G are injectable formulations.

For maternal syphilis, we calculated penicillin demand using antenatal care coverage and syphilis positivity rates, assuming two doses of benzathine penicillin G 2.4 million IU per case. 

We estimated penicillin demand for congenital syphilis using WHO-recommended weight-based neonatal dosing of penicillin G (50 000 IU per kg per dose every 12 hours for the first 7 days, then every 8 hours for the next 3 days)^1^ and converted this to vial-equivalent demand based on the commonly procured 1 million IU vial strength using an average daily requirement of 0.5 million IU. 

For rheumatic heart disease, we applied prevalence estimates to calculate annual prophylaxis needs, assuming 17.3 doses of benzathine penicillin G 2.4 million IU or 730 tablets of penicillin V 250 mg per adult patient.^2^ We defined paediatric rheumatic heart disease dosing assumptions but excluded them from the analysis due to insufficient age-disaggregated data. We used Institute for Health Metrics Evaluation estimates (2021) for rheumatic heart disease and data from the WHO Global Health Observatory for syphilis.^14^^,^^19^^,^^20^

We reviewed all data submitted by the nine countries for internal consistency, and cross-checked unclear or implausible values with country focal points and supporting documentation where possible. We treated unresolved values as missing, with no imputation performed. 

## Results

### Registration and medicines list status

Product registration and inclusion in the national essential medicine list of the five penicillin formulations vary across the nine countries. Benzathine penicillin G 1.2 million IU is registered in all countries except Timor-Leste and is included in seven national essential medicines lists, with expedited or waived access in Bhutan, Maldives, Thailand and Timor-Leste. Benzathine penicillin G 2.4 million IU is registered in five countries, all nine include it on their national essential medicines lists, with prioritization in Bhutan and Thailand.

Penicillin G is also broadly registered (5/9 countries) and included in some national essential medicines lists (4/9 countries), with prioritization in Bhutan and Maldives. Penicillin V 250 mg is similarly widely registered (8/9 countries) and included in multiple national essential medicines lists (8/9 countries), with Bhutan offering priority access. Penicillin V 500 mg shows minimal registration (4/9 countries) and limited national essential medicines list inclusion (4/9 countries) across the region (Table 3).

**Table 3:** Penicillin product registration and inclusion status in national essential medicines lists, WHO South-East Asia Region, 2025

Member State	Benzathine penicillin G 1.2 million IU			Benzathine penicillin G 2.4 million IU			Penicillin G 1 million IU			Penicillin V 250 mg			Penicillin V 500 mg	
	Registration	In national essential medicines list		Registration	In national essential medicines list		Registration	In national essential medicines list		Registration	In national essential medicines list		Registration	In national essential medicines list
Bangladesh	Yes	Yes		Yes	Yes		Yes	No		Yes	Yes		Yes	Yes
Bhutan	Yes	No		Yes	Yes		No	No		Yes	Yes		No	No
India	Yes	Yes		Yes	Yes		Yes^a^	Yes^a^		Yes	Yes		Yes	No
Maldives	Yes	Yes		Yes	Yes		No	No		Yes	Yes		Yes	Yes
Myanmar	Yes	No		Yes	Yes		Yes	No		Yes	Yes		Yes	Yes
Nepal	Yes	Yes		No	Yes		No	Yes		Yes	Yes		No	No
Sri Lanka	Yes	Yes		No	No		Yes	No		Yes	No		No	No
Thailand	Yes	Yes		No	Yes		Yes	Yes		Yes	Yes		No	Yes
Timor-Leste	No	Yes		No	Yes		No	Yes		No	Yes		No	No

IU: international units; WHO: World Health Organization.

^a^ Penicillin G 0.5 million IU is registered and included in the national essential medicines list in India instead of 1 million IU.

### Manufacturing and patent status

Manufacturing capacity for penicillin formulations varies across the region (Table 4). Benzathine penicillin G 1.2 million IU is manufactured in Bangladesh, India, Nepal and Sri Lanka, while the 2.4 million IU formulation is manufactured in Bangladesh and India. Penicillin G is manufactured in India, Sri Lanka and Thailand. Production of penicillin V 250 mg is similarly widespread, with local manufacturing reported in five countries. Penicillin V 500 mg is manufactured exclusively in India. None of the penicillin formulations are protected by patents.

**Table 4:** Local manufacture of penicillin products used for syphilis and rheumatic heart disease, WHO South-East Asia Region, 2025^a ^

Member State	Benzathine penicillin G 1.2 million IU	Benzathine penicillin G 2.4 million IU	Penicillin G	Penicillin V 250 mg	Penicillin V 500 mg
Bangladesh	Square	Square	No local manufacturer	No local manufacturer	No local manufacturer
Bhutan	No local manufacturer	No local manufacturer	No local manufacturer	No local manufacturer	No local manufacturer
India	Karnataka Antibiotics & Pharmaceuticals Limited	Karnataka Antibiotics & Pharmaceuticals Limited	Jolly Pharmacy; Karnataka Antibiotics & Pharmaceuticals Limited	Hindustan Antibiotics Limited	Hindustan Antibiotics Limited
Maldives	No local manufacturer	No local manufacturer	No local manufacturer	No local manufacturer	No local manufacturer
Myanmar	No local manufacturer	No local manufacturer	No local manufacturer	Myanmar Pharmaceutical Industrial Enterprise	No local manufacturer
Nepal	National Health Care	No local manufacturer	No local manufacturer	Nepal Pharmaceutical Laboratories Pvt.Ltd	No local manufacturer
Sri Lanka	Navesta	No local manufacturer	Navesta	State Pharmaceuticals Manufacturing Corporation	No local manufacturer
Thailand	No local manufacturer	No local manufacturer	General Drugs House Co., Ltd	Medicpharma Co., Ltd	No local manufacturer
Timor-Leste	No local manufacturer	No local manufacturer	No local manufacturer	No local manufacturer	No local manufacturer

IU: international units; WHO: World Health Organization.

^a^ None of the five penicillin products listed are patented in the country of manufacture.

### Procurement and donation mechanism

Procurement mechanisms across the region are diverse and external donation support remains limited (Table 5). Benzathine penicillin G 1.2 million IU is procured in most countries through either centralized or mixed systems, with mixed approaches in Bangladesh, Bhutan and Thailand and fully centralized procurement in Maldives and Sri Lanka. Only Bangladesh reported donations of benzathine penicillin G 1.2 million IU, received through targeted programmes for syphilis and rheumatic heart disease. 

**Table 5:** Procurement system and donation status of penicillin products, WHO South-East Asia, 2022–2024

Member State	Benzathine penicillin G 1.2 million IU		Benzathine penicillin G 2.4 million IU		Penicillin G		Penicillin V 250 mg		Penicillin V 500 mg	
	Procurement system	Donation status	Procurement system	Donation status	Procurement system	Donation status	Procurement system	Donation status	Procurement system	Donation status
Bangladesh	Mixed	Rare, targeted donations; predominantly self-procured	Not procured	No donation	Not procured	No donation	Procured only in private sector	No donation	Not procured	No donation
Bhutan	Mixed	No donation	Not procured	No donation	Not procured	No donation	Not procured	No donation	Not procured	No donation
India	Mixed	No donation	Centralized	No donation	Not procured	No donation	Not procured	No donation	Not procured	No donation
Maldives	Mixed	No donation	Centralized	No donation	Centralized	No donation	Centralized	No donation	Not procured	No donation
Myanmar	Mixed	No donation	Not procured	No donation	Mixed	No donation	Not procured	No donation	Not procured	No donation
Nepal	Decentralized	No donation	Decentralized	FHI 360 donation (2024)	Decentralized	No donation	Decentralized	No donation	Not procured	No donation
Sri Lanka	Centralized	No donation	Centralized	No donation	Centralized	No donation	Centralized	Medical Supply Division donation (2022)^a^	Centralized	No donation
Thailand	Mixed (hospital-based)	No donation	Not procured	No donation	Not procured	No donation	Not procured	No donation	Not procured	No donation
Timor-Leste	Centralized	No donation	Centralized	WHO donation (2024)	Centralized	WHO donation (2024)	Not procured	No donation	Centralized	No donation

IU: international units; WHO: World Health Organization.

^a^ Medical Supply Division is a government organization responsible for providing pharmaceuticals.

Four of the reporting countries procure benzathine penicillin G 2.4 million IU through centralized systems. Nepal uses a decentralized approach and also received a donation from FHI 360 and WHO in 2024. Five countries procure penicillin G; four through centralized or mixed arrangements while Nepal relies on decentralized procurement. Timor-Leste received donations from WHO for benzathine penicillin G 2.4 million IU and penicillin G in 2024. 

Maldives and Sri Lanka procure penicillin V 250 mg through a centralized approach. Sri Lanka also reported a donation facilitated by the government’s Medical Supply Division in 2022. Penicillin V 500 mg has the least information, with confirmed centralized procurement only in Sri Lanka and Timor-Leste. India is planning decentralized procurement through state-level units, including state-acquired immunodeficiency syndrome control societies for procurement related to sexually transmitted infections.

### Product availability and shortages

Table 6 presents data on product availability and shortages across Member States for the period 2022–2024. Benzathine penicillin G 1.2 million IU was consistently available in Myanmar and Sri Lanka; the Maldives and Thailand reported shortages related to meeting routine programmatic demand, as reflected in country stock and supply; Timor-Leste reported delays and four countries did not report on stockout. Benzathine penicillin G 2.4 million IU was sufficient in Sri Lanka; five countries reported shortages while three countries did not report. Most countries (6/9) did not report on the stockout of penicillin G and only Myanmar reported adequate procurement. Penicillin V 250 mg was sufficient only in Sri Lanka, although an interruption occurred during 2022 due to economic crisis and five countries did not report. For penicillin V 500 mg, data were limited; only Thailand and Timor-Leste reported supply and both indicated inadequate supply.

**Table 6:** Availability and shortages of penicillin formulations, WHO South-East Asia Region, 2022–2024

Member State	Benzathine penicillin G 1.2 million IU			Benzathine penicillin G 2.4 million IU			Penicillin G			Penicillin V 250 mg			Penicillin V 500 mg	
	Procurement adequacy	Stock-out (shortage)		Procurement adequacy	Stock-out (shortage)		Procurement adequacy	Stock-out (shortage)		Procurement adequacy	Stock-out (shortage)		Procurement adequacy	Stock-out (shortage)
Bangladesh	NR	NR		NR	No		NR	NR		NR	NR		NR	NR
Bhutan	NR	NR		No	No		NR	NR		No	No		NR	NR
India	NR	NR		No	No		NR	NR		NR	NR		NR	NR
Maldives	No	Yes		No	Yes		NR	NR		NR	NR		NR	NR
Myanmar	Yes	Yes^ a^		NR	NR		Yes	NR		NR	NR		NR	NR
Nepal	NR	No		NR	NR		NR	NR		NR	No		NR	NR
Sri Lanka	Yes	No		Yes	No		NR	NR		Yes	Yes^ b^		NR	NR
Thailand	No^ c^	Yes		No	No		No	No		No	No		No	No
Timor-Leste	No	Yes		No	Yes		No	Yes		No	Yes		No	Yes

IU: international units; NR: not reported; WHO: World Health Organization.

^a^ Due to 3 months shipment delay.

^b^ Due to economic crisis.

^c^ Due to limited local manufacturers.

### Clinical indications and use guidelines

We identified national guidance on the clinical use of penicillin across the nine Member States. Most countries include benzathine penicillin G for the treatment of syphilis and rheumatic heart disease, with additional indications, such as yaws, in some settings. 

Bangladesh, India, Myanmar, Nepal, Sri Lanka and Thailand have guidelines for benzathine penicillin G 1.2 million IU and Maldives reference it in broader guidance for sexually transmitted infections. Formal guidelines are absent in Bhutan and Timor-Leste.

Guidance for benzathine penicillin G 2.4 million IU is available in India, Myanmar, Nepal and Thailand, with partial coverage in Maldives. 

Treatment guidelines in India, Myanmar and Sri Lanka cover penicillin G; five countries cover penicillin V 250 mg and no country reported having guidelines for the 500 mg formulation.

Clinical indications also vary by country (Table 7). India, Nepal and Sri Lanka report the broadest range of penicillin uses across multiple conditions and populations, while Maldives and Myanmar focus primarily on syphilis and rheumatic heart disease. Bhutan and Timor-Leste do not specify indications or patient groups for penicillin formulations. (Table 7). 

**Table 7:** Clinical indications and inclusion in the national guidelines for penicillin formulations, WHO South-East Asia Region, 2025

Product	Country	Included in national guidelines	Indication
Benzathine penicillin G 1.2 million IU	Bangladesh	Yes	Acute otitis media; group A streptococcal infections; syphilis; diphtheria carrier prophylaxis; streptococcal impetigo; primary and secondary prophylaxis of rheumatic fever
	India	Yes	Syphilis; endemic treponematoses (yaws, bejel, pinta); streptococcal infections; primary and secondary prophylaxis of rheumatic fever and rheumatic heart disease (standard prophylactic dose, age- and weight-adjusted)
	Maldives	Partially	Syphilis
	Myanmar	Yes	Syphilis, rheumatic heart disease (secondary prophylaxis)
	Nepal	Yes	Syphilis, erysipelas; yaws; pinta; rheumatic fever; post-streptococcal glomerulonephritis
	Sri Lanka	Yes	Syphilis, limited use for streptococcal infections and prophylaxis (systemic infections primarily managed with aqueous penicillin G)
Benzathine penicillin G 2.4 million IU	India	Yes	Syphilis (adult treatment dosing); endemic treponematoses; selected streptococcal infections (not routinely used for rheumatic heart disease prophylaxis)
Penicillin G 1 million IU	India	Yes	Neurosyphilis; congenital syphilis; oto-syphilis; ocular syphilis
	Myanmar	Yes	Congenital syphilis
	Sri Lanka	Yes	Severe systemic infections including meningitis, endocarditis, and anthrax
Penicillin V 250 mg	Bangladesh	Yes	Rheumatic heart fever (alternative prophylaxis to benzathine penicillin G)
	India	Yes	Rheumatic heart disease (alternative prophylaxis)
	Myanmar	Yes	Rheumatic heart disease (alternative prophylaxis)
	Nepal	Yes	Acute otitis media; rheumatic fever
	Sri Lanka	Yes	Severe systemic infections including meningitis and endocarditis (limited use)

IU: international units; WHO: World Health Organization.

### Costs

**Fig. 1:**
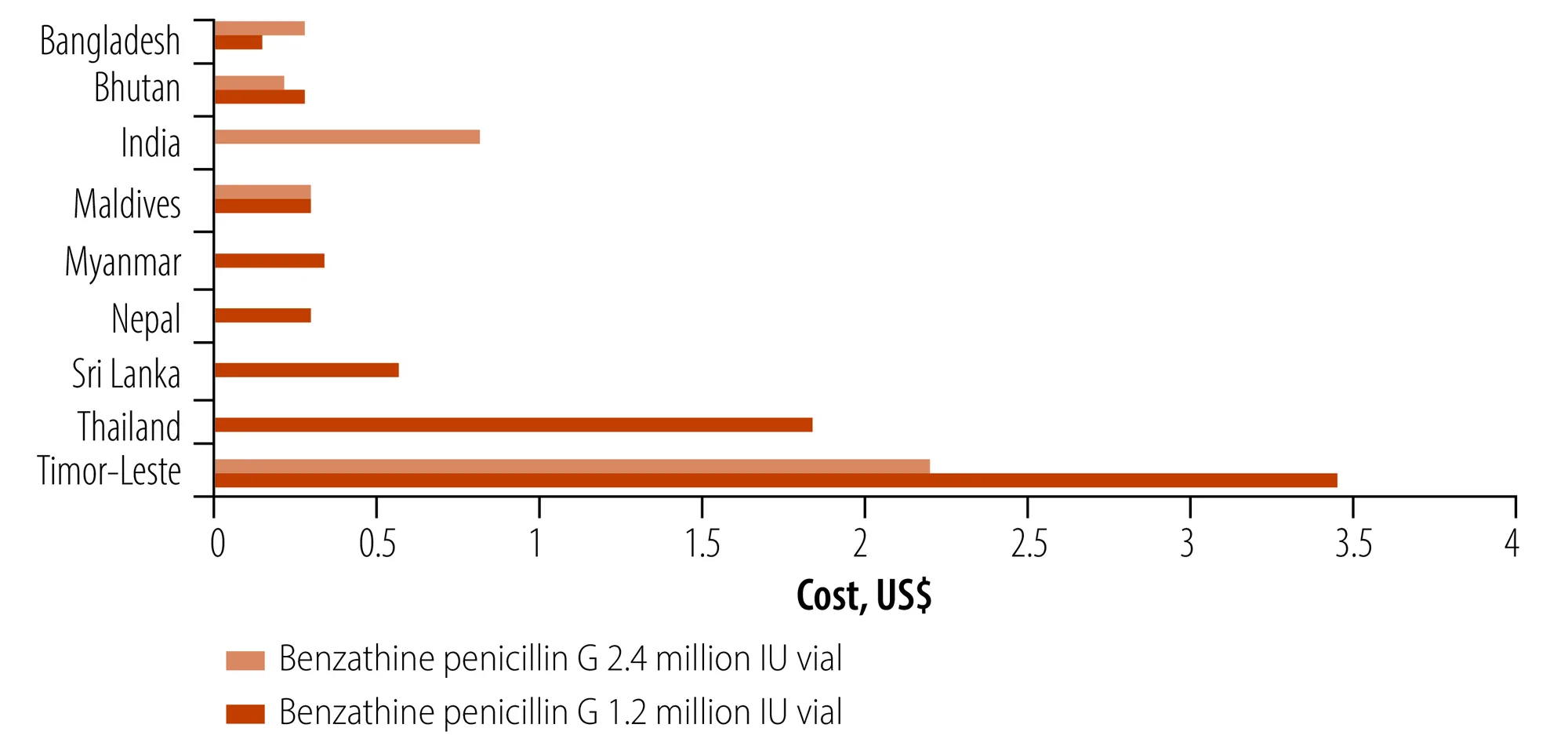
Unit cost comparison of benzathine penicillin G, WHO South-East Asia Region, 2025

As of 2025, benzathine penicillin G 1.2 million IU ranged from approximately 0.15 United States dollars (US$) in Bangladesh to US$ 1.84 in Thailand and US$ 3.45 in Timor-Leste. The 2.4 million IU formulation showed a similar pattern, with lower prices per vial in Bangladesh and Bhutan and higher prices in India (US$ 0.82) and Timor-Leste (US$ 4.10; Fig. 1).

US$: United States dollars; WHO: World Health Organization.

Note: the figure shows the unit costs for those countries that report cost data.

**Fig. 2:**
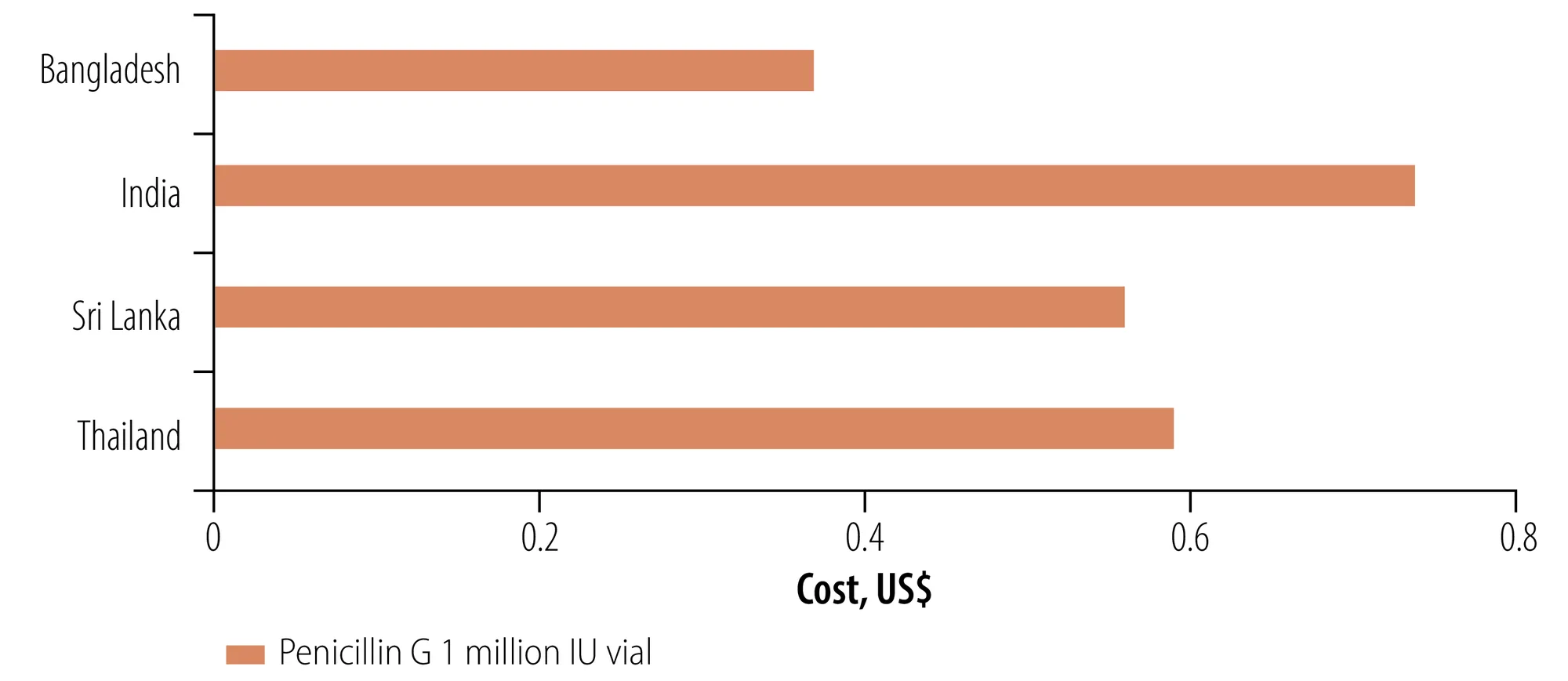
Unit cost comparison of penicillin G, WHO South-East Asia Region, 2025

**Fig. 3:**
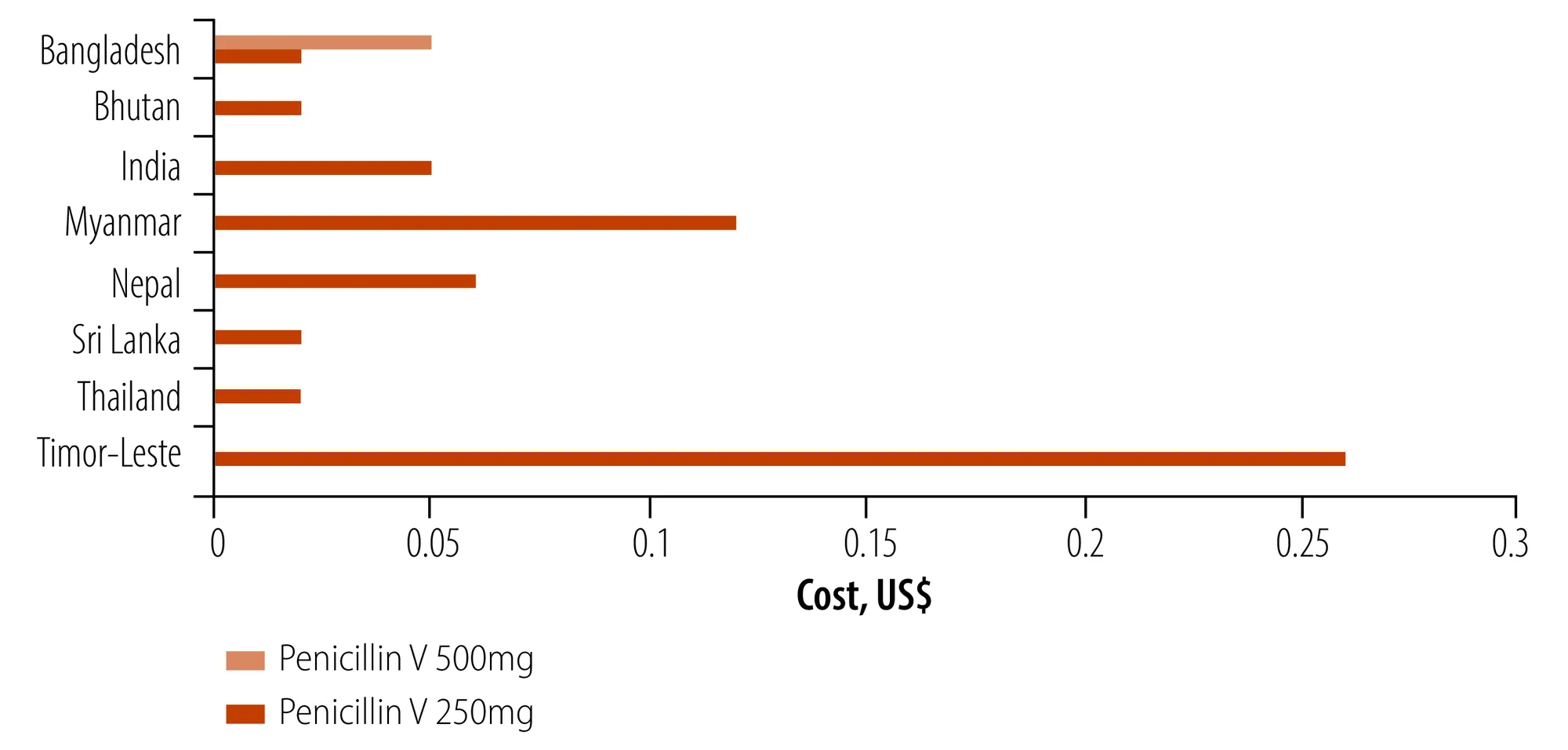
Unit cost comparison of penicillin V, WHO South-East Asia Region, 2025

Bangladesh, India, Sri Lanka and Thailand reported penicillin G prices; unit costs were similar at US$ 0.56–0.59 (Fig. 2). Penicillin V 250 mg was widely available, with prices at around US$ 0.02 per tablet reported in four countries, although Myanmar reported a higher price (US$ 0.12; Fig. 3). Penicillin V 500 mg has limited pricing data; only Bangladesh provided data (Fig. 3).

US$: United States dollars; WHO: World Health Organization.

Note: the figure shows the unit costs for those countries that report cost data.

US$: United States dollars; WHO: World Health Organization.

Note: the figure shows the unit costs for those countries that report cost data.

### Quantification of demand and surveillance mechanisms 

For 2025, quantification approaches and demand reporting varied considerably across countries and penicillin formulations, with a limited number of countries conducting formal estimation and surveillance. Where implemented, countries use a mix of facility-level reporting, programme data and consumption-level approaches. Reported annual demand varied widely, from very low volumes (25 vials of benzathine penicillin G 1.2 million IU) in Maldives to substantial requirements, including up to around 100 000 vials of benzathine penicillin G 1.2 million IU and approximately 732 000 vials of benzathine penicillin G 2.4 million IU in Sri Lanka. 

Quantification of penicillin G and penicillin V remained limited. Timor-Leste reported an annual need of approximately 2790 vials of penicillin G and 192 500 tablets of penicillin V 250 mg and Sri Lanka indicated they need approximately 15 million tablets of penicillin V 250 mg. No countries reported systems for quantifying penicillin V 500 mg. 

Surveillance systems are inconsistently applied, with structured approaches reported only in Bhutan, Myanmar and Timor-Leste. 

### Demand forecasting based on disease burden data 

Morbidity-based forecasting estimated the total demand for penicillin-based treatments at 7485 vials of penicillin G; approximately 171 966 061 vials of benzathine penicillin G 2.4 million IU for syphilis and rheumatic heart disease; and 923 million tablets of penicillin V 250 mg. These totals reflect adult demand only; we have not included demand estimates for paediatric rheumatic heart disease. The corresponding costs are US$ 13 841, US$ 12 703 and US$ 48 005 181, respectively. India leads in demand (and total annual cost burden) for benzathine penicillin G 2.4 million IU. India also has the highest demand for penicillin V 250 mg, while Bangladesh and Thailand incur the highest total annual cost for it. Detailed estimates are provided in Table 8.

**Table 8:** Morbidity-based forecasting of penicillin demand and total costs, WHO South-East Asia Region, 2025

Member State	Demand					Cost, US$			
	Penicillin G vials^a^	Benzathine penicillin G 2.4 million IU vials^b^	Benzathine penicillin G 2.4 million IU vials^c^	Penicillin V 250 mg, tablets^d^		Penicillin G^a^	Benzathine penicillin G 2.4 million IU vials^b^	Benzathine penicillin 2.4 million IU vials^c^	Penicillin V 250 mg^d^
Bangladesh	0	0	17 621 129	94 584 003		0	0	3 348 015	25 064 761
Bhutan	245	222	78 410	420 876		0	49	17 250	0
India	720	25 640	135 116 410	725 257 199		0	11 025	58 100 056	0
Maldives	0	0	54 710	293 662		0	0	16 413	0
Myanmar	0	6 997	6 343 227	34 048 202		0	0	0	0
Nepal	70	2 011	2 796 682	15 01 604		70.56	0	0	0
Sri Lanka	6 450	287	196 630	1 055 442		6849.90	0	0	591 047
Thailand	0	15 537	7 057 150	37 880 293		0	0	0	22 349 373
Timor-Leste	0	374	148 234	795 668		6920.46	1629	645 707	0
Total	7 485	51 068	171 966 061	923 053 121		13 841	12 703	62 127 441	48 005 181

IU: international unit; US$: United States dollars; WHO: World Health Organization.

^a^ Indicates demand and costs of benzathine penicillin G for congenital syphilis

^b^ Indicates demand and costs for pregnant women with syphilis

^c^ Indicates demand and costs for pregnant women with syphilis and adult rheumatic heart disease

^d^ Indicates demand and costs for adult rheumatic heart disease

Notes: WHO treatment guidelines for syphilis and rheumatic heart disease were used to estimate the demand forecasts.^1^^,^^2^ Penicillin V 500 mg was not estimated due to the lack of data provided by the countries.

## Discussion

Access to benzathine penicillin G formulations across the South-East Asia Region remains uneven, with gaps in registration, manufacturing, procurement and clinical use. This study identifies three interrelated challenges, including wide price variation across countries, inconsistent national clinical guidance and persistent supply insecurity which is linked to a highly concentrated global manufacturing base.

We found that essential formulations, including benzathine penicillin G, penicillin G and penicillin V remain vulnerable to supply disruption despite their inclusion in the *WHO Model list of essential medicines, 24th list*.^5^ Prices vary substantially across the region, reflecting fragmented procurement systems, limited supplier competition and small-volume markets. These findings are consistent with evidence that penicillin supply insecurity is driven by market consolidation,^6^^,^^21^ environmental compliance requirements and low commercial incentives,^22^ with only one WHO-prequalified finished-product manufacturer as of 2025.^23^

Furthermore, our analysis shows that regional demand for penicillin is substantially higher than previously estimated, particularly when the needs for syphilis and rheumatic heart disease are considered together. Our morbidity-based estimates, indicating that approximately 172 million vials of benzathine penicillin G 2.4 million IU are required annually across the South-East Asia Region, far exceed earlier global and regional estimates and are constrained by limited country coverage and reliance on historical procurement data.^24^^,^^25^ Notably, previous analyses from the region were largely limited to India.^26^

Also, we observed substantial variation in national guidelines and surveillance practices, highlighting inconsistencies in programme implementation. These findings align with previous reports highlighting implementation barriers, including variability in clinical guidance and delivery practices^27^ and with evidence that benzathine penicillin G is underused in antenatal care, in part due to limited provider confidence in administering injections.^28^

WHO recommends prioritizing essential penicillin formulations during shortages, improving forecasting and supporting manufacturers to maintain supply.^1^ However, prescribing patterns are shifting towards alternative therapies due to prescriber preferences and safety concerns, even though there are no clinically equivalent substitutes for first-line treatment of syphilis and rheumatic heart disease.^29^^,^^30^

Gaps in guidance and procurement further affect specific formulations. Penicillin G is frequently excluded from national guidelines and is used inconsistently across countries. Similarly, penicillin V 500 mg lacks structured quantification and monitoring systems in all countries, indicating limited data availability and weak oversight for this formulation.

Small-volume markets such as Maldives and Timor-Leste face barriers including minimum order quantities, long lead times and high per-unit costs driven by dependence on imports. In India and Nepal, penicillin prices vary by procurement modality, with centralized purchasing generally securing lower prices than decentralized procurement because of greater competition.^31^ Despite subsidies, patients often still incur substantial out-of-pocket costs for administration and transport.^32^ Data gaps persist, particularly in paediatric rheumatic heart disease dosing and penicillin V 500 mg, and few countries estimate treatment needs for maternal syphilis.

Our analysis is limited by its focus on public-sector systems, the exclusion of private-sector dynamics and medicine quality and reliance on procurement prices without adjustments for inflation or purchasing power. We based demand estimates on age-standardized prevalence; therefore, the underlying rheumatic heart disease and syphilis burden data may be underestimated. In addition, there were reporting gaps across responding countries and despite verification of non-responses we did not impute these. These shortcomings limited the available information on procurement systems and medicine availability and shortages in several countries. Nevertheless, our findings highlight the scale of regional penicillin need and the urgency of targeted procurement action amid ongoing shortages and a shrinking manufacturer base.

In conclusion, the persistent gaps in registration, procurement, pricing and guidelines undermine access to essential penicillin formulations across the South-East Asia Region. Price disparities, supply shortages and weak forecasting reflect structural market failures driven by fragmented procurement systems, limited supplier competition and insufficient policy prioritization. Addressing these challenges will require stronger procurement systems, harmonized regulatory frameworks and coordinated support for sustainable manufacturing. Regional collaboration and targeted policy action are essential to ensuring equitable access to penicillin and reducing the burden of two treatable yet persistently neglected conditions in the region.
